# Adaptive Layer-Dependent Threshold Function for Wavelet Denoising of ECG and Multimode Fiber Cardiorespiratory Signals

**DOI:** 10.3390/s25247644

**Published:** 2025-12-17

**Authors:** Yuanfang Zhang, Kaimin Yu, Chufeng Huang, Ruiting Qu, Zhichun Fan, Peibin Zhu, Wen Chen, Jianzhong Hao

**Affiliations:** 1 School of Ocean Information Engineering, Jimei University, Xiamen 361021, China; 202311810006@jmu.edu.cn (Y.Z.); 202411810021@jmu.edu.cn (C.H.); 202512854005@jmu.edu.cn (R.Q.); fanzhichun@jmu.edu.cn (Z.F.); peibin.zhu@jmu.edu.cn (P.Z.); 2 School of Mechanical Engineering and Automation, Fuzhou University, Fuzhou 350108, China; 2500210016@fzu.edu.cn; 3 Institute for Infocomm Research (I2R), Agency for Science, Technology and Research (A*STAR), Singapore 138632, Singapore

**Keywords:** autocorrelation function, layered threshold function, optical fiber sensor, ECG signal, wavelet transform

## Abstract

This paper proposes an adaptive layer-dependent threshold function (ALDTF) for denoising electrocardiogram (ECG) and multimode optical fiber-based cardiopulmonary signals. Based on wavelet transform, the method employs a layer-dependent threshold function strategy that utilizes the non-zero periodic peak (NZOPP) of the signal’s normalized autocorrelation function to adaptively determine the optimal threshold for each decomposition layer. The core idea applies soft thresholding at lower layers (high-frequency noise) to suppress pseudo-Gibbs oscillations, and hard thresholding at higher layers (low-frequency noise) to preserve signal amplitude and morphology. The experimental results show that for ECG signals contaminated with baseline wander (BW), electrode motion (EM) artifacts, muscle artifacts (MA), and mixed (MIX) noise, ALDTF outperforms existing methods—including SWT, DTCWT, and hybrid approaches—across multiple metrics. It achieves a ΔSNR improvement of 1.68–10.00 dB, ΔSINAD improvement of 1.68–9.98 dB, RMSE reduction of 0.02–0.56, and PRD reduction of 2.88–183.29%. The method also demonstrates excellent performance on real ECG and optical fiber cardiopulmonary signals, preserving key diagnostic features like QRS complexes and ST segments while effectively suppressing artifacts. ALDTF provides an efficient, versatile solution for physiological signal denoising with strong potential in wearable real-time monitoring systems.

## 1. Introduction

Real-time monitoring of cardiorespiratory signals and ensure their accuracy are crucial for the clinical diagnosis of cardiovascular diseases [1,2,3]. Among various sensing technologies, multi-mode fiber-based cardiorespiratory monitoring has attracted widespread attention due to its advantage of real-time monitoring, and resistance to electromagnetic interference, making it suitable for long-term ambulatory application [4,5]. However, both traditional electrocardiogram (ECG) signals and multi-mode fiber-optic cardiorespiratory signals are prone to noise interference during acquisition. Common noise type includes baseline wander (BW) caused by body movements, electrode motion (EM) artifacts from unstable skin-electrode contact, and muscle artifacts (MAs) generated by muscle contractions [6,7,8]—all of which distort signal morphology, obscure critical diagnostic features (e.g., QRS complexes, ST segments), and reduce the reliability of clinical analysis. Thus, numerous denoising methods have been developed, each with its own unique advantages and limitations: (1) Empirical Mode Decomposition (EMD)-based models: EMD decomposes non-stationary and non-linear signals into Intrinsic Mode Functions (IMFs) for noise separation [9]. However, it suffers from modal aliasing and endpoint effects. Subsequently, Ensemble Empirical Mode Decomposition (EEMD) has been proposed to mitigate these issues [10], but at the cost of increased computational complexity. (2) Wavelet transform-based models [11,12]: Stationary Wavelet Transform (SWT) overcomes Discrete Wavelet Transform’s (DWT) translation sensitivity, while Dual-Tree Complex Wavelet Transform (DTCWT) effectively reduces artifacts [13,14,15], but their performance heavily depends on the selection of thresholds, threshold functions and wavelet bases [16,17]. (3) Sparse representation-based models: Leveraging signal sparsity, these models separate sparse signal components from residual noise via dictionary learning and greedy algorithms [18], yet face challenges like complex hyperparameter tuning. (4) Statistical model-based methods: Using tools like Kalman filters [19], these methods estimae hidden clean signal from noisy observations, but their high computational complexity limits practical application. (5) Deep learning models: Denoising Autoencoders (DAEs) and Generative Adversarial Networks (GANs) perform well in multi-noise scenarios [20,21,22,23], but their “black-box” nature and high resource demand hinder deployment on wearable devices [24]. (6) Hybrid models: Combining multiple techniques to address single-method limitations—e.g., VMD-EWT for power-line interference and baseline wander removal [25], and DWT-ADTF for complex noise [26]—but they increase computational complexity. In summary, wavelet-based technology is particularly favored for wearable real-time heartbeat monitoring due to its simplicity and effectiveness, but its performance is highly influenced by threshold, threshold function, decomposition layer, and wavelet basis. Building on our previous work, Yu et al. [27] addressed the problem of selecting accurate layer-dependent thresholds to distinguish signal from noise coefficients. Zhu et al. [28] subsequently improved upon by refining the threshold function. Despite this improvement, its core limitation remained the use of a monolithic threshold function across all decomposition layers. This paper achieves a critical leap forward by proposing an adaptive layer-dependent threshold function (ALDTF), which dynamically selects the optimal threshold function according to the characteristics of the wavelet coefficients at each decomposition layer.

The proposed ALDTF operates on the following principle: wavelet coefficients in the lower decomposition layers typically contain detailed components and more high-frequency noise, with relatively small amplitudes. For such coefficients, the soft threshold function is more suitable, as it effectively suppresses pseudo-Gibbs oscillations through continuous shrinkage while introducing only minimal constant deviations to the signal components. In contrast, coefficients in the higher decomposition layers primarily represent the signal’s contour and low-frequency noise, exhibiting larger amplitudes. For these coefficients, the hard threshold function is more appropriate, as it avoids introducing constant bias, thereby better preserving signal amplitude and preventing waveform distortion.

Based on the accurate distinction method of signals and noise proposed in reference [27], ALDTF further achieves fine control over low-level oscillations and high-level deviations through a hierarchical threshold function strategy. This approach strikes an effective balance between “thorough noise suppression” and “maximal retention of key waveform features, such as the QRS complex and ST segment.” Guided by the principle that signals exhibit correlation whereas noise does not, the proposed method adaptively determines the most suitable threshold function for the wavelet coefficients at each layer, thus enabling effective separation of signal and noise components.

The objective of this study is to develop a universal denoising framework compatible with both conventional and emerging sensing modalities. To demonstrate its generality and robustness, the framework was evaluated on ECG signals and cardiorespiratory signals acquired via multi-mode fiber sensors. For quantitative validation, the denoising performance of the proposed method was assessed on ECG signals corrupted by baseline wander (BW), electrode motion (EM), muscle artifact (MA), and mixed (MIX) noise, and compared with three categories of existing approaches: (1) threshold-based methods [27]; (2) methods combining thresholding with improved threshold functions [28]; (3) advanced techniques, including DWT, SWT, DTCWT, empirical wavelet transform (EWT), tunable Q-factor wavelet transform (TQWT) and hybrid methods (VMD + DFA + DWT, VMD + DWT, EMD + DWT) [29,30,31]. Further validation was performed using ECG signals from the PTB database [32], as well as experimentally measured cardiorespiratory signals. Finally, the application potential of the proposed technique and promising future research directions are discussed.

## 2. Method of Layered Threshold Functions

**Figure 1:**
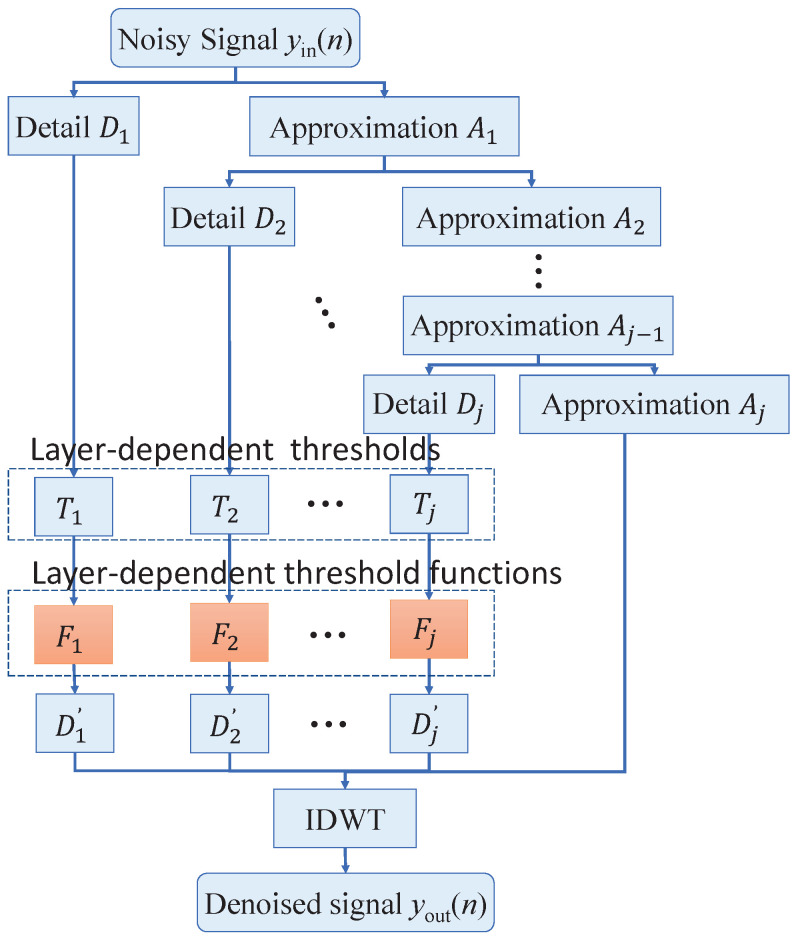
Principle of the layer-dependent threshold functions.

The advantage of this threshold function over traditional threshold functions is that it selects an appropriate threshold function for each layer based on the signal and noise distribution characteristics of that layer, as illustrated in Figure 1. The noisy signal $y_{in}\left(n\right)$ is decomposed into detail coefficients $D_{1},D_{2},\ldots ,D_{j}$ and approximation coefficients $A_{j}$. After distinguishing between signal and noise components in the detail coefficients using thresholds $T_{1},T_{2},\ldots ,T_{j}$, the coefficients representing noise are processed with a threshold function $F_{1},F_{2},\ldots ,F_{j}$ to obtain $D_{1}^{\prime },D_{2}^{\prime },\ldots ,D_{j}^{\prime }$. Finally, an inverse transform is performed to yield the denoised signal $y_{out}\left(n\right)$.

The hierarchical threshold function can be expressed as follows:

(1) $${\hat{\omega }}_{i}^{j}=\mathrm{sgn}\left(\omega_{i}^{j}\right)\cdot \left|\omega_{i}^{j}\right|/2\cdot \left\{\tanh\left[\alpha^{j}\left(\right|\omega_{i}^{j}\left|\;-\;\lambda^{j}\right)\right]\;+\;1\right\},$$

where $\omega_{i}^{j}$ and ${\hat{\omega }}_{i}^{j}$ denote the wavelet coefficients with decomposition level *j* before and after processing, respectively. $\alpha^{j}$ is the tuning parameter for the *j*th layer, which enables the hierarchical threshold function to transition smoothly between soft and hard threshold functions.

In this study, the biorthogonal 6.8 (bior 6.8) wavelet was employed for the wavelet decomposition of both ECG and multimode fiber cardiorespiratory signals. The bior 6.8 wavelet was selected due to its properties of symmetry and near-linear phase response, which are crucial for preserving the morphological features of non-stationary biomedical signals like the QRS complex and ST segment in ECG [33,34]. Its analysis and synthesis filters are specifically designed to minimize distortion during the reconstruction process [35]. Furthermore, the bior 6.8 wavelet offers an effective trade-off between smoothness and compact support, making it highly suitable for capturing the transient characteristics of cardiorespiratory signals while effectively separating them from noise across different decomposition layers [36].

Algorithm 1 outlines the binary interpolation algorithm for selecting the optimal tuning factor. Its core logic is as follows: by evaluating the quality of the denoised signal, it reversely determines the suitability of the adopted tuning factor, thereby accurately identifying the optimal tuning factor. The evaluation metric employed is the non-zero periodic peak value (NZOPP) of the normalized autocorrelation function (NACF) of the denoised signal [27].

(2) $$\mathrm{NACF}\left(k\right)=\frac{\sum_{n=0}^{N-k-1}y_{\mathrm{out}}\left(n\right)y_{\mathrm{out}}\left(n+k\right)}{\sqrt{\sum_{n=0}^{N-1}y_{\mathrm{out}}^{2}\left(n\right)\sum_{n=0}^{N-k-1}y_{\mathrm{out}}^{2}\left(n+k\right)}},$$

where *N* is the signal length, *n* denotes the sampling index, and *k* is the lag parameter (k=0, 1, …, N−1). This indicator outperforms traditional evaluation indicators in that it does not require accurate noise levels or clean reference signals, which are typically unavailable in practical measurements.

Traditional evaluation metrics, including Signal-to-Noise Ratio (SNR), Signal-to-Noise and Distortion Ration (SINAD), Root Mean Square Error (RMSE), Percentage Root Mean Square Difference (PRD), are defined as below. Notably, all these metrics require a clean reference signals x(n) for calculation.

(3) $$\mathrm{SNR}=10\log_{10}\sum_{n=1}^{N}\frac{{\left[x\left(n\right)\right]}^{2}}{{\left[y_{\mathrm{out}}\left(n\right)-x\left(n\right)\right]}^{2}},$$

(4) $$\mathrm{RMSE}=\sqrt{\frac{1}{N}\left(\sum_{n=1}^{N}{\left[x\left(n\right)-y_{\mathrm{out}}\left(n\right)\right]}^{2}\right)},$$

(5) $$\mathrm{PRD}=\sqrt{\sum_{n=1}^{N}\frac{|x\left(n\right)-y_{\mathrm{out}}{\left(n\right)|}^{2}}{x{\left(n\right)}^{2}}}\times 100\%,$$

(6) $$\mathrm{SIN}\mathrm{AD}=10\cdot \log_{10}\left(\frac{P_{s}}{P_{n}+P_{d}}\right),$$

in which $P_{s}$, $P_{n}$, and $P_{d}$ are power of signal, noise and denoised signal, respectively.

(7) $$P_{s}=\frac{1}{N}\sum_{n=0}^{N-1}{\left|x\left(n\right)\right|}^{2},\;P_{n}=\frac{1}{N}\sum_{n=0}^{N-1}|y_{\mathrm{out}}{\left(n\right)-x\left(n\right)|}^{2},\;P_{d}=\frac{1}{N}\sum_{n=0}^{N-1}{\left|y_{\mathrm{out}}\left(n\right)-y_{\mathrm{in}}\left(n\right)\right|}^{2}.$$

**Algorithm 1** Binary Search for Optimal Tuning Factors**Require:** Maximize NZOPP**Ensure:** Optimal tuning factor $\alpha_{\mathrm{opt}}$ with precision $10^{-6}$  1: Initialize $\left[\alpha_{\mathrm{start}},\alpha_{\mathrm{end}}\right]=\left[0,\mathrm{Max}(\mathrm{coefficient})\right]$  2: **while**
$\alpha_{\mathrm{end}}-\alpha_{\mathrm{start}}>10^{-6}$
**do**  3:    $\alpha_{\mathrm{mid}}=\frac{\alpha_{\mathrm{start}}+\alpha_{\mathrm{end}}}{2}$  4:    $\alpha_{\mathrm{left}}=\frac{\alpha_{\mathrm{start}}+\alpha_{\mathrm{mid}}}{2}$  5:    $\alpha_{\mathrm{right}}=\frac{\alpha_{\mathrm{mid}}+\alpha_{\mathrm{end}}}{2}$  6:    Compute NZOPP values:  7:       $N_{\mathrm{start}}=\mathrm{NZOPP}\left(\alpha_{\mathrm{start}}\right)$  8:       $N_{\mathrm{left}}=\mathrm{NZOPP}\left(\alpha_{\mathrm{left}}\right)$  9:       $N_{\mathrm{mid}}=\mathrm{NZOPP}\left(\alpha_{\mathrm{mid}}\right)$10:       $N_{\mathrm{right}}=\mathrm{NZOPP}\left(\alpha_{\mathrm{right}}\right)$11:       $N_{\mathrm{end}}=\mathrm{NZOPP}\left(\alpha_{\mathrm{end}}\right)$12:    Find Max(N)=$\max(N_{\mathrm{start}}$, $N_{\mathrm{left}}$, $N_{\mathrm{mid}}$, $N_{\mathrm{right}}$, $N_{\mathrm{end}})$13:    **if** Max(N) is in $\left[N_{\mathrm{start}},N_{\mathrm{mid}}\right]$ **then**14:        let $\alpha_{\mathrm{end}}=\alpha_{\mathrm{mid}}$15:    **else if**
Max(N) is in $\left[N_{\mathrm{mid}},N_{\mathrm{end}}\right]$
**then**16:        let $\alpha_{\mathrm{start}}=\alpha_{\mathrm{mid}}$17:    **else if**
Max(N) is in $\left[N_{\mathrm{left}},N_{\mathrm{right}}\right]$
**then**18:        let $\alpha_{\mathrm{start}}=\alpha_{\mathrm{left}}$ and $\alpha_{\mathrm{end}}=\alpha_{\mathrm{right}}$19:    **end if**20: **end while**21: **return**
$\alpha_{\mathrm{opt}}=\frac{\alpha_{\mathrm{start}}+\alpha_{\mathrm{end}}}{2}$

## 3. Materials, Experimentations and Analysis

To validate the effectiveness of the proposed layer-dependent threshold function, we applied it to denoise ECG signals contaminated by various noises. First, we compared its performance with our recently proposed layer-dependent threshold and an improved non-layer-dependent threshold function. We then extended the comparison to traditional state-of-the-art methods, including SWT, DTCWT, EWT, TQWT, VMD + DFA + DWT, VMD + DWT, and EMD + DWT. Deep learning methods were excluded due to their incompatibility with real-time processing on wearable devices.

**Figure 2:**
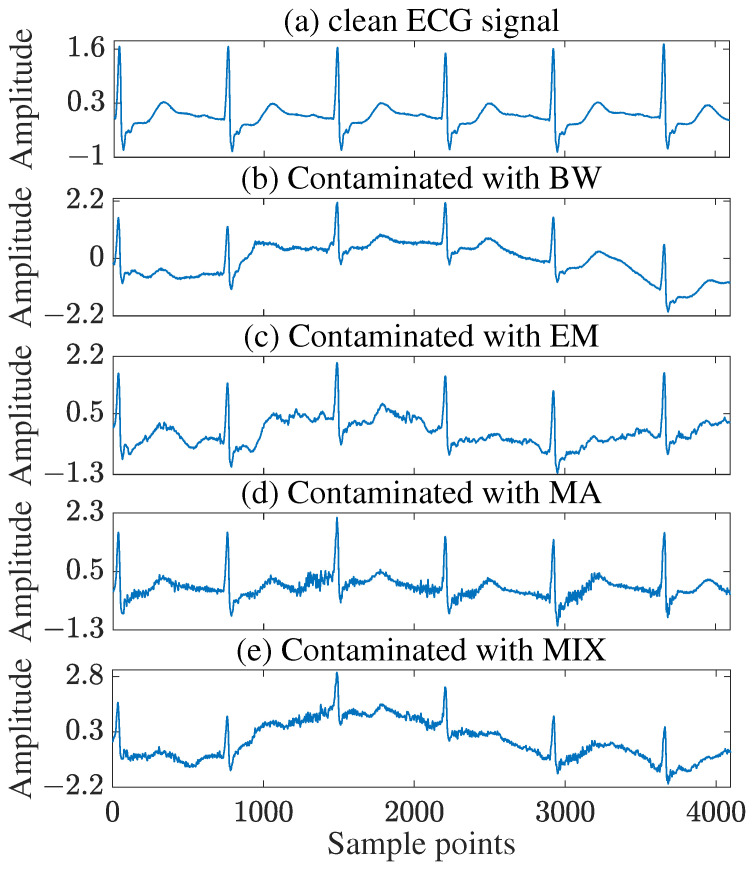
ECG signal s0010rem (**a**) clean and contaminated with (**b**) BW, (**c**) EM, (**d**) MA, and (**e**) MIX.

Test signals were selected from two established databases. The PTB Diagnostic ECG Database [32] provided s0010rem, s0016lrem, and s0026lrem, which contain data from myocardial infarction patients with varied infarction locations (e.g., inferolateral, anterior) and comorbidities (e.g., diabetes mellitus, hyperlipoproteinemia), representing typical cardiovascular disease profiles. The Creighton University Ventricular Tachyarrhythmia Database [37] contributed cu07 and cu11, which include complete recordings of sustained ventricular tachycardia, ventricular flutter, and ventricular fibrillation episodes, capturing the full progression from normal rhythm to critical arrhythmias. These signals are particularly valuable for evaluating algorithm performance under acute pathological conditions. Noise sources from the MIT-BIH Noise Stress Test Database included BW, EM, MA, and mixed noise (MIX) [38]. As an example, Figure 2 shows the s0010rem ECG signal contaminated by these noise types.

All experiments were conducted using MATLAB R2023a on a Windows 10 system with an Intel Core i9 processor and 32 GB of RAM.

### 3.1. Validation of Layer-Dependent Wavelet Threshold Function

**Figure 3:**
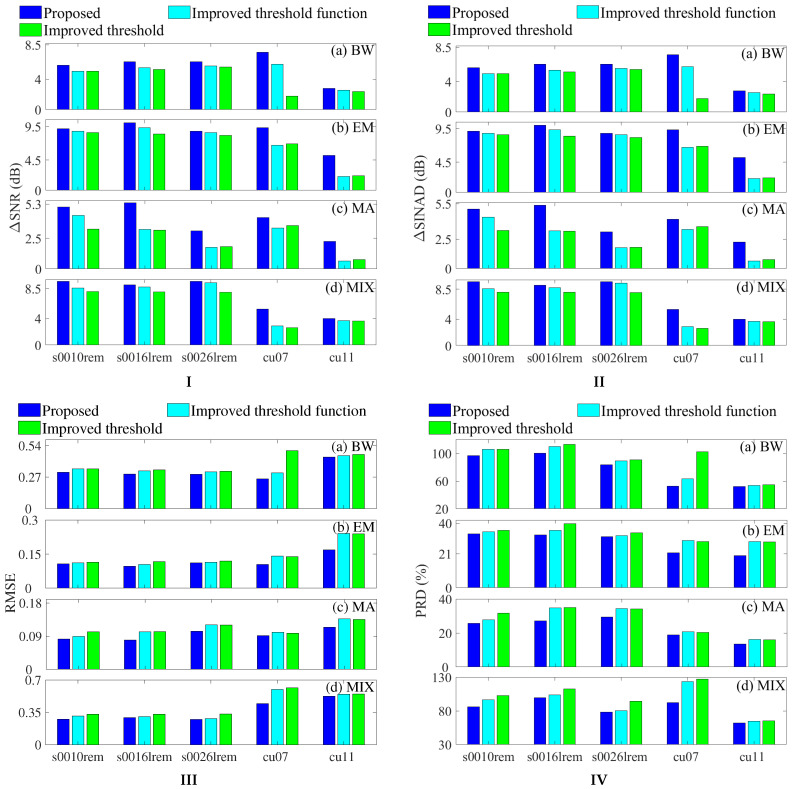
Comparison of (**I**) ΔSNRs, (**II**) ΔSINADs, (**III**) RMSEs, and (**IV**) PRDs obtained by improved threshold [27], improved threshold function [28], and the proposed method (ALDTF) for noise reduction in several types of ECG signals containing different noises, including (**a**) BW, (**b**) EM, (**c**) MA, and (**d**) MIX.

Figure 3 compares the denoising performance of three methods applied to noisy ECG signals. Our previously proposed hierarchical thresholding method [27] has already demonstrated significant advantages over existing techniques, while reference [28] further improved the denoising performance on this basis. The ALDTF method proposed in this paper builds upon these advances and achieves even better denoising results. Consequently, among all the methods evaluated, the proposed ALDTF achieves the maximum ΔSNR (Output SNR − Input SNR) and maximum ΔSINAD (Output SINAD − Input SINAD), while simultaneously yielding the minimum RMSE and minimum PRD. For instance, when dealing with the s0016lrem ECG signal contaminated by BW noise, the ΔSNR (refer to Supplementary Material S1) rises from 5.2543 dB (improved threshold alone) to 5.4831 dB (combination of improved threshold and non-layered threshold function) and finally to 6.2710 dB (combination of improved threshold and layer-dependent threshold function). Meanwhile, the RMSE decreases from 0.3319 to 0.3233 and then to 0.2952. The results show that using an improved threshold to accurately distinguish the signal and noise components of each layer’s wavelet coefficients, and further adaptively applying a layered threshold function to process the wavelet coefficients based on the signal characteristics of each layer, can effectively reduce the signal oscillation of the low decomposition layer and the constant deviation of the high decomposition layer’s signal.

### 3.2. Validation for Noisy ECG Signal

**Figure 4:**
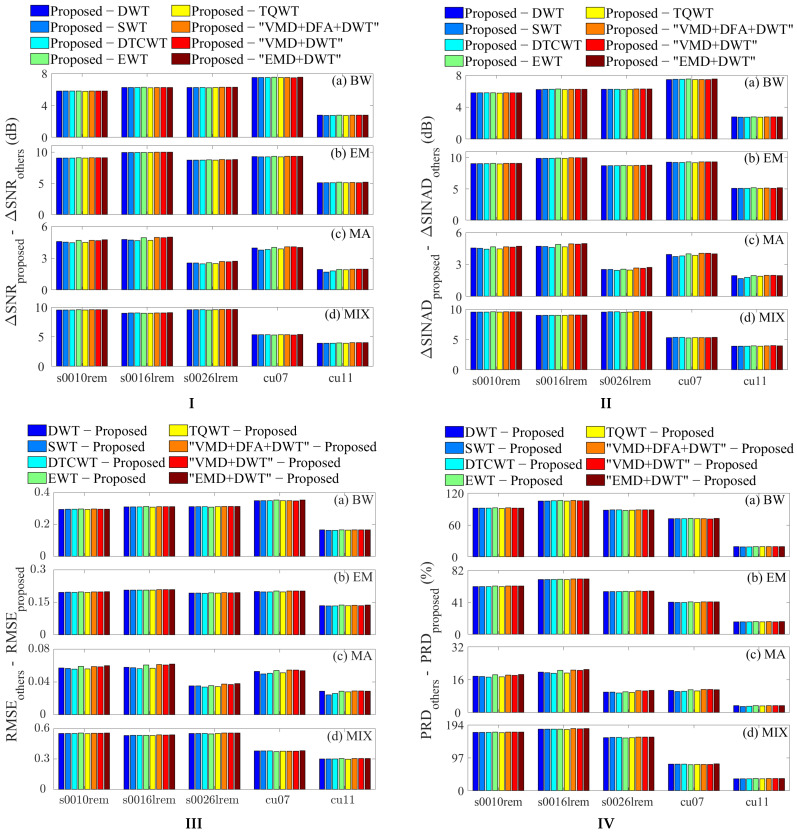
Comparison of the differences in (**I**) ΔSNRs, (**II**) ΔSINADs, (**III**) RMSEs, and (**IV**) PRDs between the proposed method and existing denoising techniques for ECG signals contaminated with different noise types: (**a**) BW, (**b**) EM, (**c**) MA, and (**d**) MIX.

Figure 4 presents a performance comparison of various denoising methods (detailed data are provided in Supplementary Material S2). The bar charts clearly illustrate the performance differences between the proposed ALDTF method and the other approaches:

In Figure 4I,II, the bars indicate the extent of improvement in ΔSNR and ΔSINAD achieved by ALDTF over the other methods; In Figure 4III,IV, the bars represent the performance gap between the other methods and ALDTF in terms of RMSE and PRD.

This difference-based representation was adopted because plotting the absolute values of the evaluation metrics would render the bars of the other methods too small to distinguish. Such a presentation maintains visual clarity while effectively highlighting the superior performance of ALDTF compared to the other methods.

Specifically, for ECG signals contaminated by BW, EM, MA, and MIX noise, the proposed method achieves ΔSNR values that are 2.73–7.54 dB, 5.08–10.00 dB, 1.68–4.99 dB, and 3.91–9.62 dB higher than those of existing techniques (DWT, SWT, DTCWT, etc.), respectively (Figure 4I). Correspondingly, its ΔSINAD values are 2.73–7.54 dB, 5.08–9.98 dB, 1.68–4.96 dB, and 3.91–9.62 dB higher, respectively (Figure 4II). In terms of error metrics, the proposed method exhibits lower RMSE values by 0.16–0.35, 0.13–0.21, 0.02–0.06, and 0.30–0.56 compared to existing methods (Figure 4III), and lower PRD values by 19.29–106.37%, 15.87–71.07%, 2.88–20.98%, and 35.38–183.29%, respectively (Figure 4IV). These results fully demonstrate that the proposed method not only suppresses noise more thoroughly but also minimizes deviations from the clean signal and preserves key ECG features (e.g., QRS complexes) more completely.

**Figure 5:**
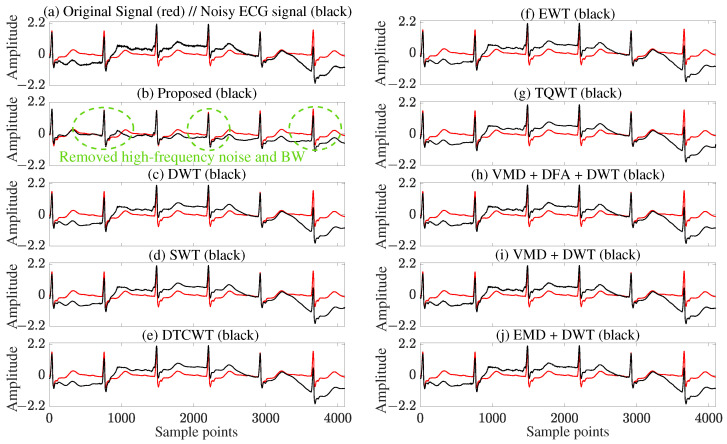
Denoising results for the s0010rem ECG signal with BW noise: (**a**) Original clean (red) and noisy (black) signals; (**b**) Proposed method; (**c**) Conventional DWT; (**d**) SWT; (**e**) DTCWT; (**f**) EWT; (**g**) TQWT; (**h**) VMD + DFA + DWT; (**i**) VMD + DWT; (**j**) EMD + DWT.

To intuitively evaluate the fidelity of the denoised signals in Figure 4, Figure 5 showcases the denoising results of various methods, using the s0010rem electrocardiogram signal contaminated with BW noise as an example. As shown in Figure 5b, the proposed method effectively eliminates high-frequency noise and baseline drift (marked by the green dashed circle) while retaining critical features such as signal amplitude and QRS waveform morphology. In contrast, other methods (Figure 5c–j) suffer from residual noise or amplitude attenuation, which may compromise the accuracy of clinical diagnosis.To observe the computational efficiency of different methods, Table 1 compares their computational costs. The proposed method takes 17.6 s, which is lower than VMD+DFA+DWT (32.4 s) and higher than other wavelet methods (DWT, SWT, DTCWT, etc). Therefore, the proposed method achieves the best denoising effect while maintaining real-time performance.

### 3.3. Validation for Measured ECG Signal

**Figure 6:**
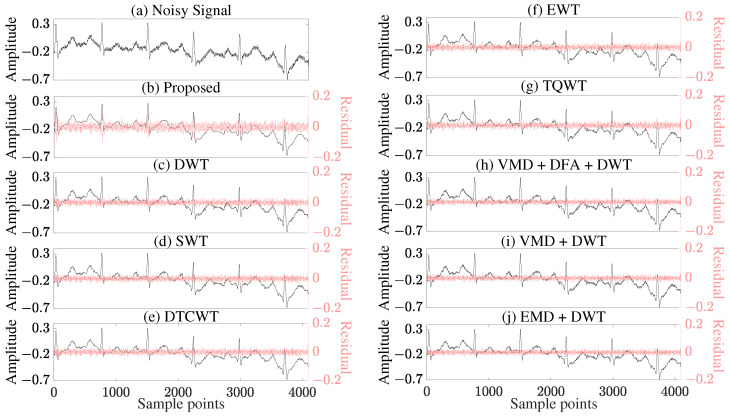
Visual comparison of (**a**) measured signal and denoising results of (**b**) Proposed method, (**c**) Conventional DWT, (**d**) SWT, (**e**) DTCWT, (**f**) EWT, (**g**) TQWT, (**h**) VMD + DFA + DWT, (**i**) VMD + DWT, (**j**) EMD + DWT.

To further validate the practical applicability of the proposed method, Figure 6 demonstrates its denoising performance on a measured ECG signal. Due to the absence of a true clean reference signal, quantitative evaluation is not feasible. Therefore, residual curves (i.e., the difference between the noisy and denoised signals) are superimposed to enhance the visual distinguishability of the results. ﻿

In terms of denoising effectiveness, the 4-layer decomposition outperforms the 3-layer one, as evidenced by its larger residual curve amplitude, indicating the removal of more noise components. Although the 5-layer decomposition yields a residual amplitude similar to that of the 4-layer, its denoised waveform exhibits distortion (see Supplementary Material S3 for details). Overall, the 4-layer decomposition achieves the cleanest denoised signal with the proposed method (Figure 6b). Compared with other methods (Figure 6c–j), the proposed approach demonstrates superior performance in both noise suppression and waveform preservation. While the comparison methods show inadequate noise suppression, the proposed method not only removes noise more thoroughly but also maintains the complete waveform characteristics of the signal (Figure 6b).

### 3.4. Validation for Multi-Mode Fiber Heartbeat and Respiration Signal

**Figure 7:**
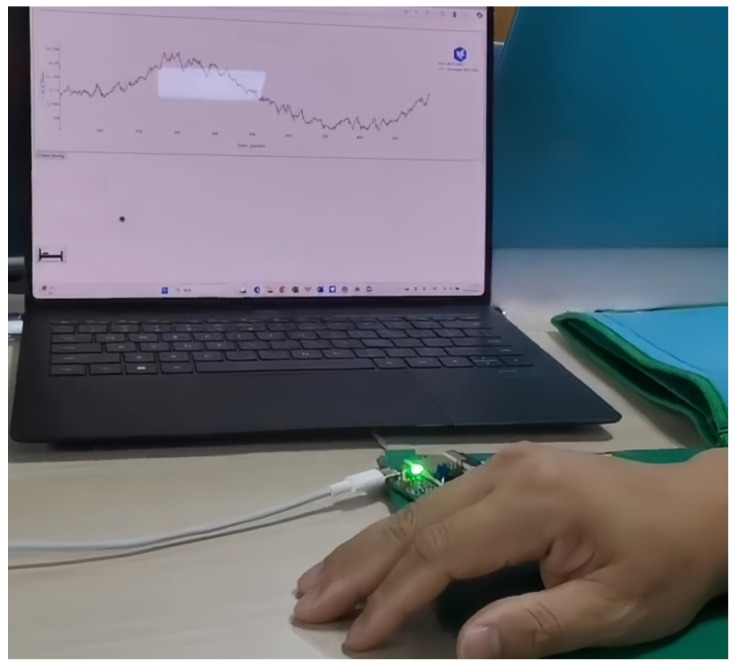
Optical fiber vital sign monitoring system.

The multi-mode optical fiber cardiopulmonary sensing system is illustrated in Figure 7. A laser with a wavelength of 1310 nm, generated by a TOSA-LC 1310 LED laser (manufactured by Accelink Technologies Co., Ltd. and sourced from Wuhan, China), is injected into a 1 m long multi-mode fiber (MMF). When the human body undergoes micro-disturbances such as heartbeat, respiration, or body movement, the light intensity distribution in the optical fiber changes accordingly. Subsequently, an OS-PD55x photodetector (manufactured by Thorlabs Inc. and sourced from Newton, MA, USA) receives these changes and converts them into a digital signal with a sampling rate of 2048 Hz. The ESP32 chip (manufactured by Espressif Systems (Shanghai) Co., Ltd. and sourced from Shanghai, China) processes the digital signal and transmits it to a computer via a Wi-Fi module, where cardiopulmonary information is displayed.

To evaluate the generalizability of the proposed algorithm in complex scenarios, we applied it to heartbeat and respiration signals acquired via multi-mode optical fiber. These signals originate from complex optical mode interference, resulting in waveform characteristics fundamentally different from those of traditional contact sensors (e.g., ECG, respiratory belts). Furthermore, the absence of a synchronous, gold-standard reference signal makes quantitative assessment infeasible. Given these challenges, we employed a qualitative visual assessment method. This approach involves a direct comparison of the signal waveforms before and after denoising, with a focus on analyzing the effectiveness of noise suppression for physiological features.

**Figure 8:**
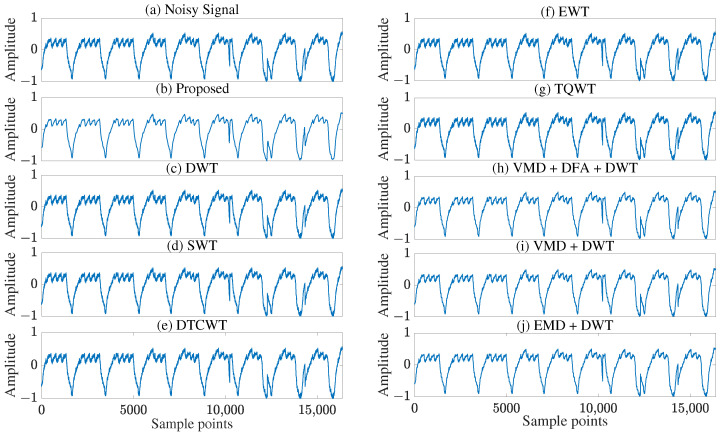
Visual comparison of (**a**) multi-mode fiber heartbeat and respiration signal and denoising results of (**b**) Proposed method, (**c**) Conventional DWT, (**d**) SWT, (**e**) DTCWT, (**f**) EWT, (**g**) TQWT, (**h**) VMD + DFA + DWT, (**i**) VMD + DWT, (**j**) EMD + DWT.

As shown in Figure 8, the proposed method achieved optimal performance with 5-layer decomposition, producing the cleanest denoised signal (Figure 8b). This outcome is attributed to the improved balance between noise removal and signal feature preservation at this level, as detailed in Supplementary Material S4. In Figure 8, the cardiopulmonary signal in Figure 8b exhibits the lowest noise and highest clarity, with an overall waveform morphology significantly superior to the denoising results in Figure 8c–j. This advantage stems from the proposed method’s ability to accurately distinguish between signal and noise components in wavelet coefficients. Specifically, it effectively reduces the oscillation of wavelet coefficients in low decomposition layers while minimizing the constant deviation of wavelet coefficients in high decomposition layers, tailored to the characteristics of signal and noise in each decomposition layer. In contrast, although Figure 8h–j show lower visual noise levels, residual noise is still clearly observable. Therefore, the denoising results for actual multi-mode optical fiber cardiopulmonary signals demonstrate that the proposed method outperforms other existing techniques.

## 4. Results

This study introduces an ALDTF for denoising ECG and multimode fiber-optic cardiorespiratory signals. Tests on ECG signals corrupted with BW, EM, MA, and MIX show that ALDTF surpasses existing methods (SWT, DTCWT, and hybrid techniques) in performance:

- ΔSNR improvement: 1.68–10.00 dB;
- ΔSINAD gain: 1.68–9.98 dB;
- RMSE reduction: 0.02–0.56;
- PRD reduction: 2.88–183.29%. ﻿

The method also proves effective on real measured ECG and fiber-optic cardiorespiratory signals, enhancing signal quality significantly. Its layer-wise adaptation handles varying noise distributions: preserving QRS complexes in high-frequency layers and ST-segment morphology in low-frequency layers while suppressing artifacts and pseudo-Gibbs oscillations.

## 5. Discussion

The proposed ALDTF method represents a significant advance in wavelet-based denoising through its dynamic, layer-specific thresholding mechanism, which effectively overcomes the rigidity of conventional approaches. By adaptively tuning thresholds for each decomposition layer, ALDTF achieves notable improvements in both SNR and RMSE while successfully addressing diverse noise types—from high-frequency muscle artifacts to low-frequency baseline wander—all while preserving essential ECG waveform features. This adaptive capability stands in stark contrast to traditional wavelet methods that apply uniform thresholding across all layers, often resulting in residual noise in lower layers or over-smoothing of critical morphological features in higher layers. The consistent superiority of ALDTF across multiple evaluation metrics and noise conditions underscores its robustness and practical versatility.

When contextualized within the current landscape of denoising research, ALDTF demonstrates distinct advantages across several methodological domains. Compared to advanced wavelet variants like DTCWT [15] that focus on transform complexity while maintaining fixed thresholding strategies, ALDTF preserves the computational simplicity of standard DWT while achieving superior performance through its intelligent, layer-specific adaptation. This makes it particularly suitable for real-time wearable systems where computational efficiency is paramount. Similarly, while hybrid decomposition-denoising models such as VMD-EWT [25] and VMD+DWT [30] can effectively target specific noise components, they often suffer from high computational complexity and mode selection challenges. ALDTF achieves comparable or superior denoising performance within a unified and computationally more efficient framework, eliminating the need for separate decomposition steps.

The comparison with data-driven deep learning models reveals another key advantage of the ALDTF approach. Despite the impressive performance of denoising autoencoders [22] and GANs [23] in handling complex noise patterns, their black-box nature, substantial data requirements, and computational demands limit their practical deployment in resource-constrained environments. ALDTF offers a transparent, model-based alternative that requires no training while delivering robust performance across different subjects and recording conditions. Furthermore, when compared to other adaptive thresholding techniques, ALDTF’s novelty lies in its dual adaptation mechanism—optimizing not only the threshold values but also the shape of the threshold function itself, enabled by the NZOPP metric derived from the denoised signal’s autocorrelation. This finer level of control allows for more nuanced preservation of signal components across different scales, resulting in significantly reduced distortion.

The primary implication of this work is that substantial denoising improvements can be achieved through refined thresholding mechanisms within established wavelet frameworks, rather than relying exclusively on more complex transforms or external models. ALDTF’s ability to preserve diagnostically critical features such as QRS complexes and ST segments with minimal distortion positions it as a valuable tool for both clinical and ambulatory ECG analysis. However, several limitations warrant consideration. The optimal number of decomposition layers was determined empirically for specific signal types, suggesting the need for an automated, signal-dependent parameter selection method to enhance generalizability. While ALDTF has been validated on both conventional ECG and novel fiber-optic cardiorespiratory signals, its performance on other biomedical signals like EEG and EMG remains to be investigated. Additionally, the computational cost of the current binary search implementation, though manageable for this study’s scope, requires further optimization for ultra-low-power wearable hardware.

Looking forward, several research directions emerge naturally from these findings. Algorithm optimization should focus on developing more efficient search strategies for the optimal tuning factors to enable real-time processing on embedded systems. The framework’s broader applicability could be extended to other quasi-periodic physiological signals such as PPG and EEG, leveraging the adaptive principles demonstrated in this work. We have also expanded the discussion to more clearly outline specific clinical scenarios where ALDTF could offer substantial benefits: in ambulatory ECG monitoring, its computational efficiency and independence from clean reference signals make it particularly suitable for long-term wearable applications; in remote patient monitoring, its robustness against common artifacts (BW, EM, MA) ensures reliable signal quality in unsupervised home environments; and for diagnostic assistance, its effectiveness in preserving key diagnostic waveforms (QRS complexes, ST segments) proves crucial for subsequent automated or manual detection of conditions such as myocardial ischemia or arrhythmias. Finally, the integration of ALDTF’s principles with deep learning architectures—either as preprocessing layers or as components of loss functions—represents a promising avenue for combining the strengths of model-based and data-driven approaches, potentially opening new pathways for synergistic denoising solutions that balance performance with interpretability and computational efficiency.

## 6. Conclusions

This study proposes the ALDTF method for wavelet-based denoising of ECG and multimode fiber-optic cardiorespiratory signals, operating without the need for clean reference signals. Departing from conventional approaches that employ fixed threshold functions uniformly across decomposition layers, ALDTF dynamically adapts both thresholds and threshold functions at different levels. This adaptability enables more precise noise suppression while effectively preserving clinically critical signal features. Experimental validation demonstrates that ALDTF significantly outperforms several existing methods in key metrics—including SNR, SINAD, RMSE, and PRD—when processing ECG signals contaminated by diverse noise types. The method also exhibits excellent denoising performance for fiber-optic cardiorespiratory signals.

In summary, ALDTF establishes a robust methodological foundation for physiological signal denoising, showing notable performance improvements over conventional techniques. While these results highlight its strong potential for clinical translation, the direct clinical impact of ALDTF requires further validation through dedicated subsequent studies. Future work will focus on: (1) collaborating with clinical partners to evaluate the method in larger and more diverse patient cohorts; (2) rigorously assessing the extent to which ALDTF-enhanced signal quality improves diagnostic accuracy in real-world settings; and (3) exploring integration pathways with clinical-grade wearable devices and remote patient monitoring platforms. Through these efforts, the practical clinical value of ALDTF can be systematically established and its adoption in healthcare settings accelerated.

## Figures and Tables

**Table 1:** Computational cost comparison.

Methods	Proposed	DWT	SWT	DTCWT	EWT	TQWT	VMD + DFA + DWT	VMD + DWT	EMD + DWT
Cost (s)	17.6268	0.0657	1.0352	0.1499	0.5598	0.3440	32.3740	4.6079	0.3689

## Data Availability

The ECG data used in this study were obtained from PhysioNet (https://physionet.org/), a publicly available research resource providing open-access biomedical signals. As all data are de-identified and pre-collected for non-commercial research purposes, no additional ethical approval was required for this secondary analysis.

## Supplementary Materials

The following supporting information can be downloaded at: https://www.mdpi.com/article/10.3390/s25247644/s1, Table S1: Comparison of ΔSNRs obtained by the proposed method, improved threshold function, and improved threshold for noise reduction of several types of ECG signals containing different noises, including (a) BW, (b) EM, (c) MA, and (d) MIX; Table S2: Comparison of ΔSINADs obtained by the proposed method, improved threshold function, and improved threshold for noise reduction of several types of ECG signals containing different noises, including (a) BW, (b) EM, (c) MA, and (d) MIX; Table S3: Comparison of RMSEs obtained by the proposed method, improved threshold function, and improved threshold for noise reduction of several types of ECG signals containing different noises, including (a) BW, (b) EM, (c) MA, and (d) MIX; Table S4: Comparison of PRDs obtained by the proposed method, improved threshold function, and improved threshold for noise reduction of several types of ECG signals containing different noises, including (a) BW, (b) EM, (c) MA, and (d) MIX. Table S5: Difference between the ΔSNRs of the proposed method and existing denoising techniques for ECG signals contaminated with different noise types: (a) BW, (b) EM, (c) MA, and (d) MIX; Table S6: Difference between the ΔSINADs of the proposed method and existing denoising techniques for ECG signals contaminated with different noise types: (a) BW, (b) EM, (c) MA, and (d) MIX; Table S7: Difference between the RMSEs of existing denoising techniques and the proposed method for ECG signals contaminated with different noise types: (a) BW, (b) EM, (c) MA, and (d) MIX; Table S8: Difference between the PRDs of existing denoising techniques and the proposed method for ECG signals contaminated with different noise types: (a) BW, (b) EM, (c) MA, and (d) MIX. Figure S1: Measured ECG signal before denoising; Figure S2: Denoising results for (I) 3-layer, (II) 4-layer, and (III) 5-layer wavelet decomposition. Panel I–III show (a) the proposed method’s performance compared to (b) DWT, (c) SWT, (d) DTCWT, (e) EWT, (f) TQWT, (g) VMD + DFA + DWT, (h) VMD + DWT, and (i) EMD + DWT. Figure S3: Multi-mode optical fiber cardiopulmonary signal before denoising; Figure S4: Denoising results for (I) 4-layer, (II) 5-layer, and (III) 6-layer wavelet decomposition. Panel I–III show (a) the proposed method’s performance compared to (b) DWT, (c) SWT, (d) DTCWT, (e) EWT, (f) TQWT, (g) VMD + DFA + DWT, (h) VMD + DWT, and (i) EMD + DWT.
