# Supplementary material for: Adaptive Layer-Dependent Threshold Function for Wavelet Denoising of ECG and Multimode Fiber Cardiorespiratory Signals
Source: Sensors (Basel). 2025 Dec 17;25(24):7644. doi: 10.3390/s25247644 (PMC12737139; doi:10.3390/s25247644)
Supplement: Supplementary file 1 [file sensors-25-07644-s001.zip › Supplementary Material/Supplementary Material S1.pdf]

Supplementary Material S1

Description: The proposed method was compared against both the improved threshold and improved threshold function approaches. Quantitative performance assessment was conducted using the following metrics: △SNR (Output SNR – Input SNR), △SINAD (Output SINAD – Input SINAD), RMSE, and PRD.

Table S1. Comparison of △SNRs obtained by the proposed method, improved threshold function, and improved threshold for noise reduction of several types of ECG signals containing different noises, including (a) BW, (b) EM, (c) MA, and (d) MIX.

Noise	ECG	Proposed	Improved Threshold Function	Improved Threshold
(a) BW	s0010rem	**5.8247**	5.0485	5.0516
	s0016lrem	**6.2710**	5.4831	5.2543
	s0026lrem	**6.2875**	5.7325	5.5802
	cu07	**7.5417**	5.9515	1.7778
	cu11	**2.7864**	2.5378	2.3659
(b) EM	s0010rem	**9.1429**	8.7997	8.5932
	s0016lrem	**10.0436**	9.3251	8.3852
	s0026lrem	**8.8265**	8.6034	8.1867
	cu07	**9.3500**	6.7231	6.9000
	cu11	**5.1961**	2.0884	2.1463
(c) MA	s0010rem	**5.0769**	4.3882	3.2540
	s0016lrem	**5.4021**	3.2202	3.1723
	s0026lrem	**3.1096**	1.7735	1.8107
	cu07	**4.1942**	3.3425	3.5562
	cu11	**2.2535**	0.6447	0.7565
(d) MIX	s0010rem	**9.6077**	8.5834	8.0584
	s0016lrem	**9.0850**	8.7373	8.0275
	s0026lrem	**9.6341**	9.4057	7.9846
	cu07	**5.4240**	2.8604	2.6293
	cu11	**3.9917**	3.6679	3.6301

Table S2. Comparison of △SINADs obtained by the proposed method, improved threshold function, and improved threshold for noise reduction of several types of ECG signals containing different noises, including (a) BW, (b) EM, (c) MA, and (d) MIX.

Noise	ECG	Proposed	Improved Threshold Function	Improved Threshold
(a) BW	s0010rem	**5.8231**	5.0472	5.0503
	s0016lrem	**6.2686**	5.4820	5.2526
	s0026lrem	**6.2868**	5.7322	5.5796
	cu07	**7.5383**	5.9495	1.7775
	cu11	**2.7864**	2.5377	2.3660
(b) EM	s0010rem	**9.1264**	8.7868	8.5788
	s0016lrem	**10.0176**	9.3112	8.3682
	s0026lrem	**8.8211**	8.5993	8.1810

Noise	ECG	Proposed	Improved Threshold Function	Improved Threshold
	cu07	**9.3279**	6.7124	6.8893
	cu11	**5.1963**	2.0884	2.1467
(c) MA	s0010rem	**5.0580**	4.3738	3.2468
	s0016lrem	**5.3717**	3.2078	3.1600
	s0026lrem	**3.1060**	1.7723	1.8095
	cu07	**4.1808**	3.3343	3.5485
	cu11	**2.2553**	0.6481	0.7610
(d) MIX	s0010rem	**9.6053**	8.5814	8.0568
	s0016lrem	**9.0828**	8.7347	8.0253
	s0026lrem	**9.6336**	9.4050	7.9846
	cu07	**5.4240**	2.8602	2.6292
	cu11	**3.9918**	3.6680	3.6302

Table S3. Comparison of RMSEs obtained by the proposed method, improved threshold function, and improved threshold for noise reduction of several types of ECG signals containing different noises, including (a) BW, (b) EM, (c) MA, and (d) MIX.

Noise	ECG	Proposed	Improved Threshold Function	Improved Threshold
(a) BW	s0010rem	**0.3108**	0.3398	0.3397
	s0016lrem	**0.2952**	0.3233	0.3319
	s0026lrem	**0.2947**	0.3141	0.3197
	cu07	**0.2550**	0.3063	0.4952
	cu11	**0.4409**	0.4537	0.4628
(b) EM	s0010rem	**0.1070**	0.1113	0.1140
	s0016lrem	**0.0965**	0.1048	0.1168
	s0026lrem	**0.1110**	0.1139	0.1195
	cu07	**0.1045**	0.1414	0.1385
	cu11	**0.1686**	0.2411	0.2395
(c) MA	s0010rem	**0.0824**	0.0892	0.1017
	s0016lrem	**0.0794**	0.1021	0.1026
	s0026lrem	**0.1034**	0.1205	0.1200
	cu07	**0.0912**	0.1006	0.0982
	cu11	**0.1141**	0.1373	0.1355
(d) MIX	s0010rem	**0.2754**	0.3099	0.3292
	s0016lrem	**0.2925**	0.3044	0.3304
	s0026lrem	**0.2746**	0.2819	0.3320
	cu07	**0.4458**	0.5989	0.6150
	cu11	**0.5258**	0.5457	0.5481

Table S4. Comparison of PRDs obtained by the proposed method, improved threshold function, and improved threshold for noise reduction of several types of ECG signals containing different noises, including (a) BW, (b) EM, (c) MA, and (d) MIX.

Noise	ECG	Proposed	Improved Threshold Function	Improved Threshold
(a) BW	s0010rem	**96.8351**	105.8871	105.8490
	s0016lrem	**100.5219**	110.0674	113.0049
	s0026lrem	**83.7792**	89.3072	90.8874
	cu07	**52.6707**	63.2522	102.2735
	cu11	**52.2230**	53.7395	54.8132
(b) EM	s0010rem	**33.3424**	34.6863	35.5208
	s0016lrem	**32.8479**	35.6808	39.7585
	s0026lrem	**31.5548**	32.3757	33.9666
	cu07	**21.5788**	29.1995	28.6106
	cu11	**19.9643**	28.5521	28.3621
(c) MA	s0010rem	**25.6762**	27.7951	31.6722
	s0016lrem	**27.0283**	35.6808	39.7585
	s0026lrem	**29.3862**	34.2725	34.1262
	cu07	**18.8388**	20.7796	20.2745
	cu11	**13.5088**	16.2576	16.0496
(d) MIX	s0010rem	**85.8120**	96.5529	102.5685
	s0016lrem	**99.5928**	103.6605	112.4879
	s0026lrem	**78.0684**	80.1485	94.3950
	cu07	**92.0709**	123.6804	127.0146
	cu11	**62.2677**	64.6330	64.9148
