# Supplementary material for: Adaptive Layer-Dependent Threshold Function for Wavelet Denoising of ECG and Multimode Fiber Cardiorespiratory Signals
Source: Sensors (Basel). 2025 Dec 17;25(24):7644. doi: 10.3390/s25247644 (PMC12737139; doi:10.3390/s25247644)
Supplement: Supplementary file 1 [file sensors-25-07644-s001.zip › Supplementary Material/Supplementary Material S2.pdf]

Supplementary Material S2

Description: The proposed method was compared against several state-of-the-art denoising techniques. Quantitative performance assessment was conducted using the following metrics: △SNR (Output SNR – Input SNR), △SINAD (Output SINAD – Input SINAD), RMSE, and PRD.

Table S5. Difference between the △SNRs of the proposed method and existing denoising techniques for ECG signals contaminated with different noise types: (a) BW, (b) EM, (c) MA, and (d) MIX.

Noise	ECG	Proposed - DWT	Proposed - SWT	Proposed - DTCWT	Proposed - EWT	Proposed - TQWT	Proposed - "VMD+DFA+DWT"	Proposed - "VMD+DWT"	Proposed - "EMD+DWT"
(a) BW	s0010rem	5.7936	5.7968	5.7976	5.8227	5.7860	5.8200	5.8136	5.8138
	s0016lrem	6.2313	6.2378	6.2404	6.2697	6.2241	6.2622	6.2574	6.2611
	s0026lrem	6.2572	6.2630	6.2623	6.2263	6.2603	6.2852	6.2799	6.2842
	cu07	7.4846	7.4912	7.4964	7.5416	7.4853	7.4862	7.4756	7.5416
	cu11	2.7731	2.7304	2.7362	2.7860	2.7482	2.7859	2.7859	2.7859
(b) EM	s0010rem	9.0511	9.0561	9.0487	9.1072	9.0420	9.1069	9.1024	9.1159
	s0016lrem	9.9420	9.9390	9.9312	9.9552	9.9267	10.0033	9.9936	9.9991
	s0026lrem	8.7417	8.7386	8.7306	8.7664	8.7313	8.7950	8.7885	8.7984
	cu07	9.2937	9.2442	9.2406	9.3495	9.2361	9.3495	9.3496	9.3495
	cu11	5.0992	5.0812	5.0804	5.1949	5.0986	5.1615	5.0909	5.1946
(c) MA	s0010rem	4.5650	4.5272	4.4680	4.6892	4.4937	4.6713	4.6508	4.7343
	s0016lrem	4.7546	4.7125	4.6436	4.9255	4.6852	4.9561	4.9294	4.9900
	s0026lrem	2.5343	2.5388	2.4570	2.5582	2.4915	2.6807	2.6514	2.7176
	cu07	3.9539	3.7739	3.8265	4.0235	3.8625	4.0659	4.0660	4.0183
	cu11	1.9432	1.6772	1.7786	1.9432	1.8922	1.9643	1.9698	1.9524
(d) MIX	s0010rem	9.5406	9.5468	9.5430	9.5896	9.5317	9.5797	9.5761	9.5895
	s0016lrem	9.0014	9.0146	9.0105	9.0049	8.9945	9.0521	9.0493	9.0675
	s0026lrem	9.5568	9.5709	9.5657	9.5146	9.5634	9.6166	9.6127	9.6148
	cu07	5.3359	5.3510	5.3503	5.2779	5.3257	5.3260	5.3224	5.3808
	cu11	3.9126	3.9120	3.9125	3.9772	3.9076	3.9804	3.9807	3.9803
Min improvement							1.68		
Max improvement							10.00		

Table S6. Difference between the △SINADs of the proposed method and existing denoising techniques for ECG signals contaminated with different noise types: (a) BW, (b) EM, (c) MA, and (d) MIX.

Noise	ECG	Proposed - DWT	Proposed - SWT	Proposed - DTCWT	Proposed - EWT	Proposed - TQWT	Proposed - "VMD+DFA+DWT"	Proposed - "VMD+DWT"	Proposed - "EMD+DWT"
(a) BW	s0010rem	5.7920	5.7952	5.7960	5.8211	5.7843	5.8184	5.8120	5.8122
	s0016lrem	6.2288	6.2354	6.2379	6.2673	6.2216	6.2598	6.2550	6.2587
	s0026lrem	6.2565	6.2623	6.2615	6.2256	6.2596	6.2844	6.2791	6.2834
	cu07	7.4811	7.4877	7.4929	7.5382	7.4817	7.4826	7.4720	7.5382
	cu11	2.7730	2.7303	2.7361	2.7860	2.7481	2.7858	2.7859	2.7859

Noise	ECG	Proposed - DWT	Proposed - SWT	Proposed - DTCWT	Proposed - EWT	Proposed - TQWT	Proposed - “VMD+DFA+DWT”	Proposed - “VMD+DWT”	Proposed - “EMD+DWT”
(b) EM	s0010rem	9.0345	9.0396	9.0322	9.0904	9.0254	9.0904	9.0859	9.0993
	s0016lrem	9.9159	9.9129	9.9051	9.9288	9.9006	9.9772	9.9675	9.9731
	s0026lrem	8.7362	8.7331	8.7251	8.7608	8.7259	8.7896	8.7830	8.7930
	cu07	9.2710	9.2214	9.2178	9.3274	9.2134	9.3274	9.3275	9.3274
	cu11	5.0991	5.0810	5.0803	5.1950	5.0985	5.1614	5.0909	5.1948
(c) MA	s0010rem	4.5464	4.5087	4.4495	4.6707	4.4751	4.6528	4.6323	4.7158
	s0016lrem	4.7249	4.6829	4.6141	4.8938	4.6557	4.9264	4.8998	4.9600
	s0026lrem	2.5307	2.5350	2.4532	2.5535	2.4877	2.6770	2.6477	2.7139
	cu07	3.9366	3.7565	3.8089	4.0070	3.8449	4.0488	4.0490	4.0019
	cu11	1.9426	1.6755	1.7770	1.9427	1.8905	1.9643	1.9698	1.9522
(d) MIX	s0010rem	9.5381	9.5443	9.5405	9.5872	9.5293	9.5772	9.5737	9.5870
	s0016lrem	8.9992	9.0124	9.0082	9.0026	8.9922	9.0498	9.0470	9.0652
	s0026lrem	9.5562	9.5703	9.5651	9.5140	9.5628	9.6161	9.6121	9.6142
	cu07	5.3358	5.3509	5.3502	5.2777	5.3255	5.3258	5.3223	5.3807
	cu11	3.9126	3.9120	3.9125	3.9772	3.9076	3.9805	3.9807	3.9804
Min improvement						1.68			
Max improvement						9.98			

Table S7. Difference between the RMSEs of existing denoising techniques and the proposed method for ECG signals contaminated with different noise types: (a) BW, (b) EM, (c) MA, and (d) MIX.

Noise	ECG	DWT - Proposed	SWT - Proposed	DTCWT - Proposed	EWT - Proposed	TQWT - Proposed	“VMD+DFA+DWT” - Proposed	“VMD+DWT” - Proposed	“EMD+DWT” - Proposed
(a) BW	s0010rem	0.2948	0.2950	0.2950	0.2968	0.2942	0.2966	0.2961	0.2962
	s0016lrem	0.3097	0.3102	0.3103	0.3124	0.3092	0.3119	0.3115	0.3118
	s0026lrem	0.3109	0.3113	0.3113	0.3088	0.3111	0.3129	0.3125	0.3128
	cu07	0.3487	0.3491	0.3495	0.3527	0.3487	0.3488	0.3481	0.3527
	cu11	0.1658	0.1629	0.1633	0.1667	0.1641	0.1667	0.1667	0.1667
(b) EM	s0010rem	0.1964	0.1965	0.1963	0.1983	0.1960	0.1983	0.1982	0.1986
	s0016lrem	0.2066	0.2065	0.2062	0.2070	0.2060	0.2087	0.2084	0.2086
	s0026lrem	0.1926	0.1925	0.1923	0.1935	0.1923	0.1945	0.1943	0.1946
	cu07	0.2001	0.1984	0.1983	0.2021	0.1981	0.2021	0.2021	0.2021
	cu11	0.1346	0.1340	0.1340	0.1380	0.1346	0.1368	0.1343	0.1380
(c) MA	s0010rem	0.0570	0.0564	0.0554	0.0590	0.0558	0.0587	0.0584	0.0597
	s0016lrem	0.0578	0.0572	0.0561	0.0606	0.0568	0.0611	0.0606	0.0616
	s0026lrem	0.0350	0.0351	0.0338	0.0354	0.0343	0.0374	0.0369	0.0380
	cu07	0.0526	0.0496	0.0505	0.0537	0.0511	0.0545	0.0545	0.0537
	cu11	0.0286	0.0243	0.0259	0.0286	0.0278	0.0289	0.0290	0.0287
(d) MIX	s0010rem	0.5506	0.5512	0.5509	0.5553	0.5498	0.5544	0.5540	0.5553
	s0016lrem	0.5320	0.5332	0.5329	0.5323	0.5313	0.5368	0.5365	0.5383
	s0026lrem	0.5505	0.5518	0.5514	0.5465	0.5511	0.5562	0.5558	0.5560
	cu07	0.3782	0.3797	0.3796	0.3728	0.3773	0.3773	0.3770	0.3825

Noise	ECG	DWT - Proposed	SWT - Proposed	DTCWT - Proposed	EWT - Proposed	TQWT - Proposed	“VMD+DFA+DWT” - Proposed	“VMD+DWT” - Proposed	“EMD+DWT” - Proposed
	cu11	0.2992	0.2991	0.2992	0.3053	0.2987	0.3056	0.3057	0.3056
	Min reduction					0.02			
	Max reduction					0.56			

Table S8. Difference between the PRDs of existing denoising techniques and the proposed method for ECG signals contaminated with different noise types: (a) BW, (b) EM, (c) MA, and (d) MIX.

Noise	ECG	DWT - Proposed	SWT - Proposed	DTCWT - Proposed	EWT - Proposed	TQWT - Proposed	“VMD+DFA+DWT” - Proposed	“VMD+DWT” - Proposed	“EMD+DWT” - Proposed
(a) BW	s0010rem	91.8400	91.9089	91.9265	92.4718	91.6735	92.4134	92.2740	92.2778
	s0016lrem	105.4574	105.6132	105.6734	106.3716	105.2867	106.1936	106.0789	106.1663
	s0026lrem	88.4070	88.5219	88.5063	87.7953	88.4679	88.9619	88.8558	88.9414
	cu07	72.0098	72.1051	72.1792	72.8306	72.0195	72.0327	71.8811	72.8306
	cu11	19.6423	19.2894	19.3371	19.7490	19.4364	19.7475	19.7483	19.7482
(b) EM	s0010rem	61.1834	61.2384	61.1579	61.7957	61.0846	61.7932	61.7438	61.8910
	s0016lrem	70.3349	70.2995	70.2071	70.4913	70.1532	71.0656	70.9491	71.0158
	s0026lrem	54.7729	54.7420	54.6626	55.0187	54.6697	55.3047	55.2392	55.3386
	cu07	41.3300	40.9726	40.9468	41.7355	40.9145	41.7353	41.7363	41.7355
	cu11	15.9455	15.8713	15.8680	16.3432	15.9432	16.2039	15.9114	16.3421
(c) MA	s0010rem	17.7530	17.5644	17.2705	18.3782	17.3978	18.2876	18.1842	18.6080
	s0016lrem	19.6965	19.4707	19.1034	20.6249	19.3247	20.7931	20.6465	20.9801
	s0026lrem	9.9560	9.9762	9.6072	10.0643	9.7627	10.6245	10.4899	10.7951
	cu07	10.8606	10.2514	10.4282	11.0997	10.5497	11.2460	11.2465	11.0816
	cu11	3.3869	2.8774	3.0698	3.3869	3.2881	3.4281	3.4388	3.4049
(d) MIX	s0010rem	171.5697	171.7539	171.6406	173.0265	171.3073	172.7297	172.6240	173.0220
	s0016lrem	181.1440	181.5712	181.4366	181.2568	180.9205	182.7866	182.6941	183.2871
	s0026lrem	156.5250	156.9056	156.7653	155.3872	156.7030	158.1461	158.0383	158.0964
	cu07	78.1118	78.4089	78.3949	76.9800	77.9119	77.9183	77.8490	78.9948
	cu11	35.4320	35.4250	35.4304	36.1617	35.3756	36.1979	36.2007	36.1967
	Min reduction						2.88		
	Max reduction						183.29		
