# Supplementary material for: Adaptive Layer-Dependent Threshold Function for Wavelet Denoising of ECG and Multimode Fiber Cardiorespiratory Signals
Source: Sensors (Basel). 2025 Dec 17;25(24):7644. doi: 10.3390/s25247644 (PMC12737139; doi:10.3390/s25247644)
Supplement: Supplementary file 1 [file sensors-25-07644-s001.zip › Supplementary Material/Supplementary Material S3.pdf]

Supplementary Material S3

Description: Figure S1 shows the measured ECG signal in this study.

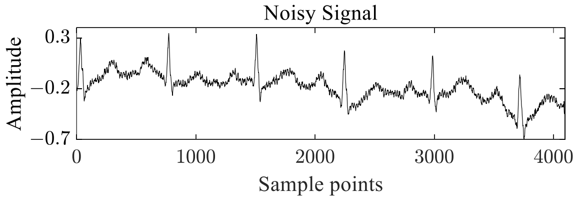

Figure S1. Measured ECG signal before denoising.

Figure S2 presents a comparison of the denoising results of the proposed wavelet method at decomposition levels of 3, 4, and 5 layers, corresponding to Figure S2 - I, Figure S2 - II, and Figure S2 - III respectively. Due to the absence of a true clean reference signal, quantitative evaluation is not feasible. Therefore, residual curves (i.e., the difference between the noisy and denoised signals) are superimposed to enhance the visual distinguishability of the results. In terms of denoising effectiveness, the 4-layer decomposition outperforms the 3-layer one, as evidenced by its larger residual curve amplitude, indicating the removal of more noise components. Although the 5-layer decomposition yields a residual amplitude similar to that of the 4-layer, its denoised waveform exhibits distortion. Consequently, the proposed method attains the cleanest denoised signal (Figure S2 - II a) with minimal residual noise and maintains signal integrity when employing 4 - layer decomposition. When contrasted with existing methods (Figure S2 - II b - i), the proposed method (Figure S2 - II a) yields a far cleaner noise reduction profile and preserves the integrity of the QRS waveform. The proposed method can accurately assess the denoising quality without relying on a clean signal reference or prior knowledge of the noise level. As a result, it can be used to determine the precise threshold and adaptive threshold function for each decomposition layer. This approach addresses the issues of poor noise reduction quality in existing methods and the incapability of real - time processing in AI - based denoising techniques. Hence, it holds promise for applications in wearable sensors.

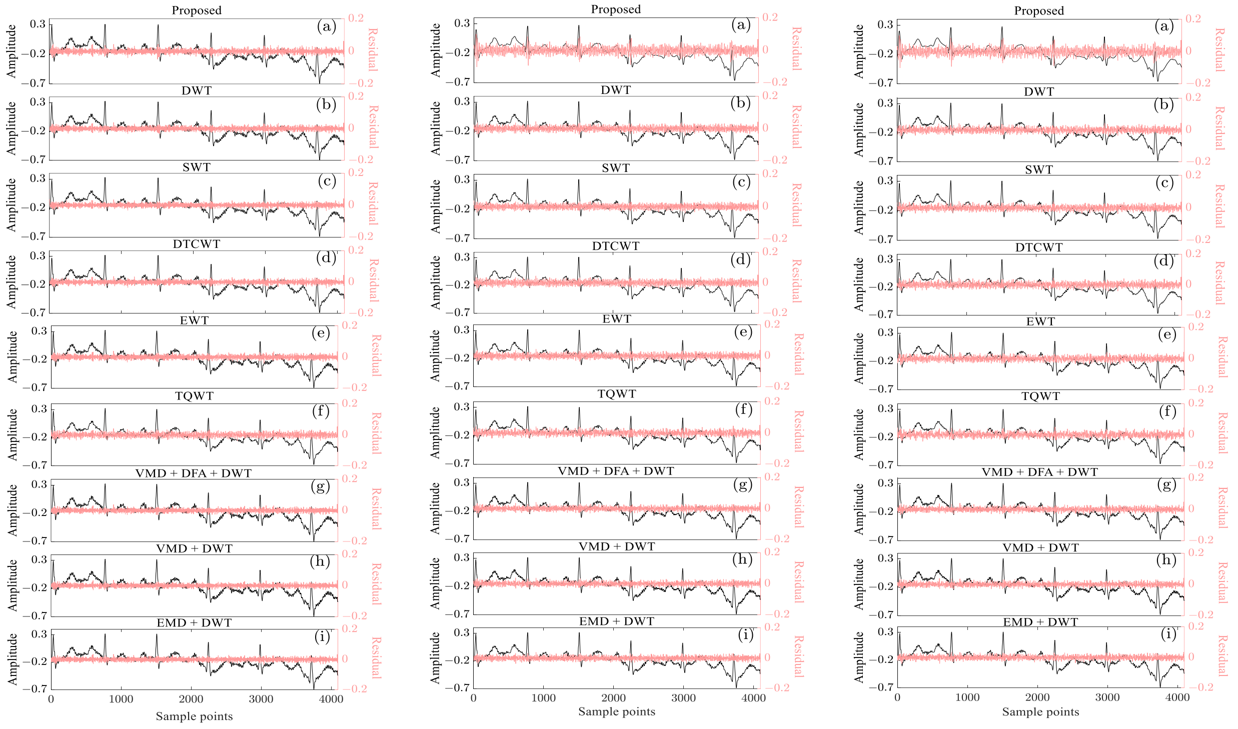

(I) 3-decomposition layer (II) 4-decomposition layer (III) 5-decomposition layer

Figure S2. Denoising results for (I) 3-layer, (II) 4-layer, and (III) 5-layer wavelet decomposition. Panel I - III

show (a) the proposed method's performance compared to (b) DWT, (c) SWT, (d) DTCWT, (e) EWT, (f) TQWT, (g) VMD+DFA+DWT, (h) VMD+DWT, and (i) EMD+DWT.
