# Supplementary material for: Adaptive Layer-Dependent Threshold Function for Wavelet Denoising of ECG and Multimode Fiber Cardiorespiratory Signals
Source: Sensors (Basel). 2025 Dec 17;25(24):7644. doi: 10.3390/s25247644 (PMC12737139; doi:10.3390/s25247644)
Supplement: Supplementary file 1 [file sensors-25-07644-s001.zip › Supplementary Material/Supplementary Material S4.pdf]

Supplementary Material S4

Description: Figure S3 shows the Multi-mode optical fiber cardiopulmonary signal in this study.

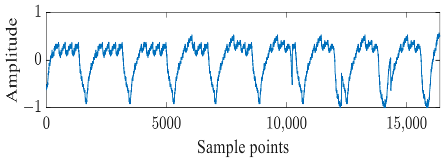

Figure S3. Multi-mode optical fiber cardiopulmonary signal before denoising.

Figure S4 shows the denoising effect of the proposed wavelet method under different decomposition levels (4, 5, and 6 layers), corresponding to Figure S4 - I, Figure S4 - II, and Figure S4 - III respectively. When the decomposition level is 4 (Figure S4 - I a), the denoised signal still contains obvious noise, indicating that this decomposition level is insufficient to achieve the optimal noise suppression. When the decomposition level increases to 6 (Figure S4 - III a), the noise is further reduced, but the signal waveform experiences a certain degree of amplitude attenuation. In contrast, when using a 5 - layer decomposition (Figure S4 - II a), the proposed method effectively suppresses the noise while maintaining the integrity of the signal, achieving the clearest denoising effect. Compared with the existing methods (Figure S4 - II b - i), the proposed method (Figure S4 - II a) not only achieves a cleaner noise suppression curve but also better retains the morphological characteristics of the QRS waveform.

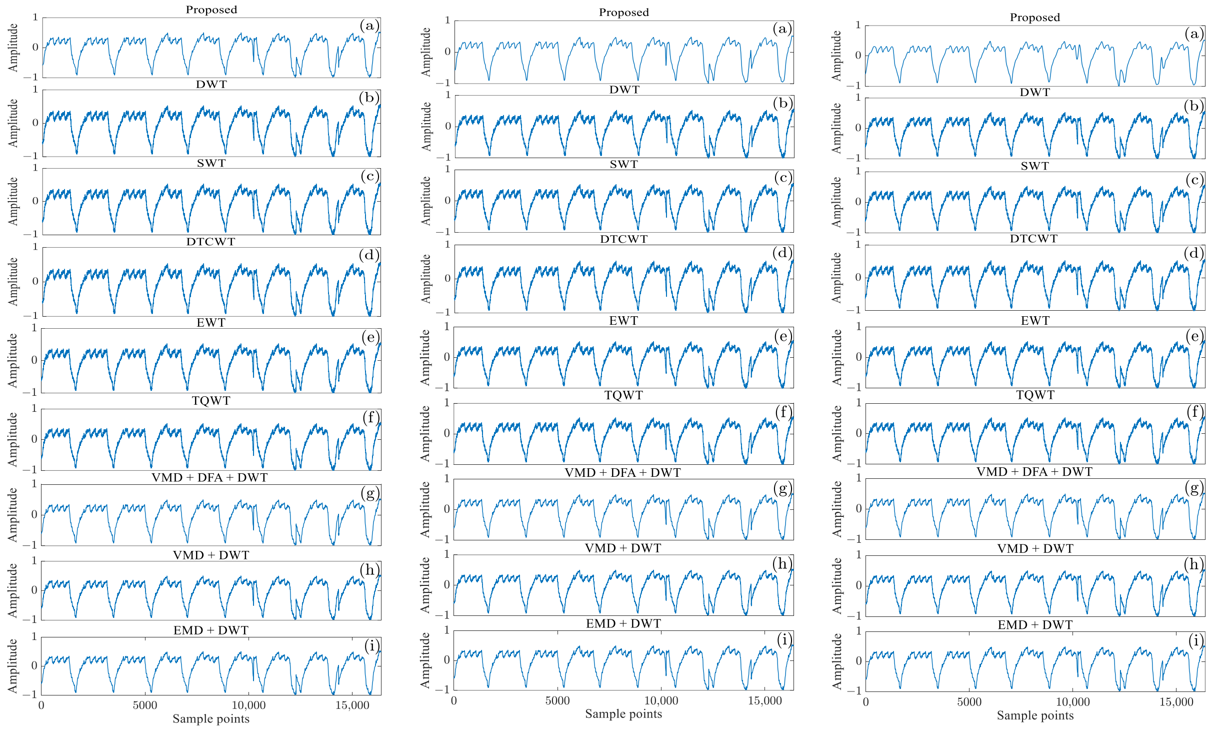

(I) 4-decomposition layer (II) 5-decomposition layer (III) 6-decomposition layer

Fig. S4. Denoising results for (I) 4-layer, (II) 5-layer, and (III) 6-layer wavelet decomposition. Panel I – III show (a) the proposed method's performance compared to (b) DWT, (c) SWT, (d) DTCWT, (e) EWT, (f) TQWT, (g) VMD+DFA+DWT, (h) VMD+DWT, and (i) EMD+DWT.
